# Effects of external body vibrations on cognitive performance through brain–cardio–respiratory dynamics

**DOI:** 10.1038/s41598-025-21639-6

**Published:** 2025-10-28

**Authors:** Masami Iwamoto, Hoshinori Kanazawa, Atsushi Narita, Shogo Yonekura, Yasuo Kuniyoshi

**Affiliations:** 1 Human Science Research-domain, Toyota Central R&D Labs., Inc, Nagakute, Japan; 2 Next Generation Artificial Intelligence Research Center, The University of Tokyo, Bunkyo City, Japan

**Keywords:** Computational biology and bioinformatics, Neuroscience

## Abstract

External body vibrations (EBV) affect the human physiological and cognitive states. Here we construct a neurophysiological model integrating a cortical model and the cardiovascular system, exploring impacts of EBV on physiological and cognitive function. We adopted a reservoir computing model for cortical dynamics to investigate cognitive effects, focusing on binocular rivalry. This model successfully replicated changes in human perceptual alternation observed during voluntary respiratory control and cycling exercise. Our simulation indicates that EBV exerts frequency-dependent effects on perceptual alternation, brain variability, and heart rate variability, demonstrating that synchronization between the locus coeruleus and the cardiorespiratory system influences these measures. These results provide mechanistic insights, implying that EBV can finely tune brain and cardiovascular dynamics, thereby potentially optimizing cognitive performance.

## Introduction

External body vibrations (EBV) influence physical and mental states in humans. Vehicle vibrations can induce sleepiness, discomfort, arousal, or comfort depending on frequency and amplitude^1–4^. EBV from earthquakes may evoke fear or anxiety, whereas not only voluntary rhythmic exercises but also involuntary EBV can reduce negative emotion and enhance positive emotion^5–7^. EBV also alter cardiorespiratory system parameters such as heart rate (HR) and respiratory rate (RR), as well as emotional or cognitive performance^8–11^.

Electroencephalography (EEG) and electrocardiography (ECG) can measure brain activity and heart performance based on various waveforms, respectively, typically when the body is at rest. EEG recording during externally applied EBV is particularly challenging because vibration-induced artefacts are often indistinguishable from intrinsic neurophysiological signals^12,13^. Recent studies have established a standard multi-stage signal-processing framework that combines head-acceleration reference signals with adaptive filtering (or ICA/ASR) techniques^14^. This approach markedly suppresses spectral overlap in the delta–beta frequency bands, thereby mitigating artefact contamination and enabling reliable EEG acquisition during oscillatory motion at 0.5–25 Hz^14^. To the best of the authors’ knowledge, however, no study has yet measured EEG under EBV at frequencies below 0.2 Hz.

Cognitive performance is related to whole-brain dynamics, depending on brain and physiological states. Better performance on a cognitive flexibility task is linked to increased brain variability (BV)^15^. BV refers to the extent to which neural activity fluctuates over time or varies from trial to trial, and it is regarded as an indicator of the flexibility of information processing. BV increased substantially during tasks compared to rest, especially in younger, faster-performing adults, while older individuals exhibited less pronounced changes^16^. Additionally, some studies have reported that BV increases with age, showing a negative correlation with reaction time variability and positive correlation with accuracy during childhood through mid-adulthood^17^. Further, Thayer et al. demonstrated that individuals with higher heart rate variability (HRV) performed better on executive-function tasks in reaction time and accuracy than those with lower HRV^18^. Therefore, collecting BV and HRV data is crucial for investigating the impact of EBV on cognitive performance.

Within the study of cognitive functions mediated by neurophysiological systems, binocular rivalry constitutes a valuable experimental paradigm. This phenomenon occurs when incompatible images presented separately to each eye induce spontaneous alternations in perceptual awareness^19^. –^20^. These stochastic alternations have made binocular rivalry a widely adopted model for investigating the neural correlates of visual awareness, attentional mechanisms, and perceptual selection^19–21^. Moreover, previous studies have demonstrated that interoceptive signals can mutually influence perceptual decision-making processes^22^.

We previously proposed a computational model to simulate the activity of the locus coeruleus (LC) - norepinephrine (NE) and cardiorespiratory systems in response to EBV, offering an alternative to experimental approaches that face challenges in measuring brain activity owing to artifact^23^. Therefore, herein, we enhanced our model by incorporating a reservoir computing (RC) approach to simulate cerebral cortex dynamics, enabling us to predict BV and HRV during EBV and thereby explore its impact on cognitive performance.

## Results

### Summary architecture of modeling

**Fig. 1:**
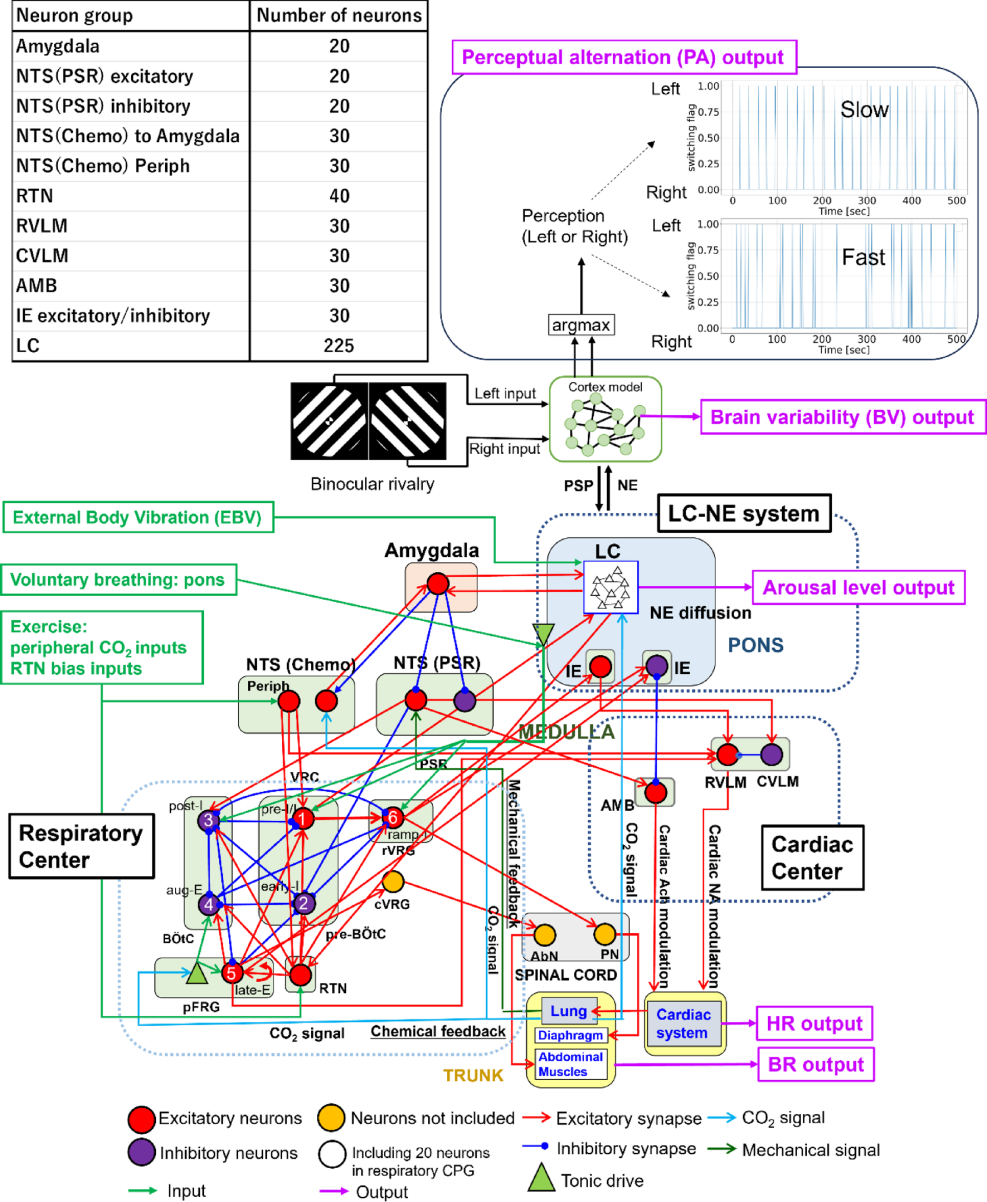
Computational model for predicting RR, HR, arousal level, and cognitive performance following external stimulation. The model includes interactions among the LC-NE, cardiorespiratory systems, and the cerebral cortex. The LC-NE and cardiorespiratory systems include interactions such as amygdala-LC interactions, the nucleus of the solitary tract (NTS), respiratory and cardiac centers, and the closed-loop respiratory cardiac systems^23^. The respiratory center and system comprise the closed-loop system for respiratory control, as proposed by Molkov et al.^25^ This closed-loop respiratory system incorporates two primary feedback pathways originating from the lungs to the respiratory central pattern generator: mechanical feedback facilitated by pulmonary stretch receptors, and chemical feedback involving peripheral chemoreceptors as well as central chemoreceptors from the retrotrapezoid nucleus (RTN) neurons. The cardiac center comprises pathways that regulate heart activity through sympathetic outflow, which modulates NE from the rostral ventrolateral medulla (RVLM), and parasympathetic outflow, which modulates acetylcholine from the ambiguous nucleus (AMB). Inspiratory-to-expiratory phase-spanning neurons (IE) in the pons integrate the activities of the respiratory and cardiac centers. Neurons were modeled using Hodgkin–Huxley (HH)-type spiking neurons. Voluntary RR is adjusted by a tonic drive parameter in the pons, whereas active expiration during exercise is regulated by inputs from peripheral CO_2_ concentrations to the NTS chemoreceptors and the RTN bias input. Vibrational inputs are directly applied to the LC. HR, RR, and NE-modulated conductance representing arousal level are outputs from the cardiac system, lungs, and LC, respectively. cVRG refers to the caudal ventral respiratory group. VRC refers to the ventral respiratory column. The cerebral cortex was modeled using RC, which receives inputs from the left and right eyes during binocular rivalry^24^. It processes these inputs to produce outputs related to perceptual alternation and brain variability. The cerebral cortex was modulated by the amount of NE from the LC, whereas the LC was modulated by postsynaptic potentials (PSP) from the cerebral cortex.

We previously proposed a computational model to simulate the activity of the LC and cardiorespiratory system elicited by EBV and investigated the effects of EBV on the RR, HR, and arousal level associated with physical risks^23^. We added a RC model simulating the dynamics of the cerebral cortex in binocular rivalry^24^ to the model and investigated the effects of EBV on cognitive performance. Figure 1 illustrates a computational model for predicting the RR, HR, arousal level, and cognitive performance following external stimulation, including two interactions: between the LC-NE and cardiorespiratory systems and between the LC-NE system and the cerebral cortex.

### Comparison of perceptual alternation for respiratory rate between model prediction and experimental data

**Fig. 2:**
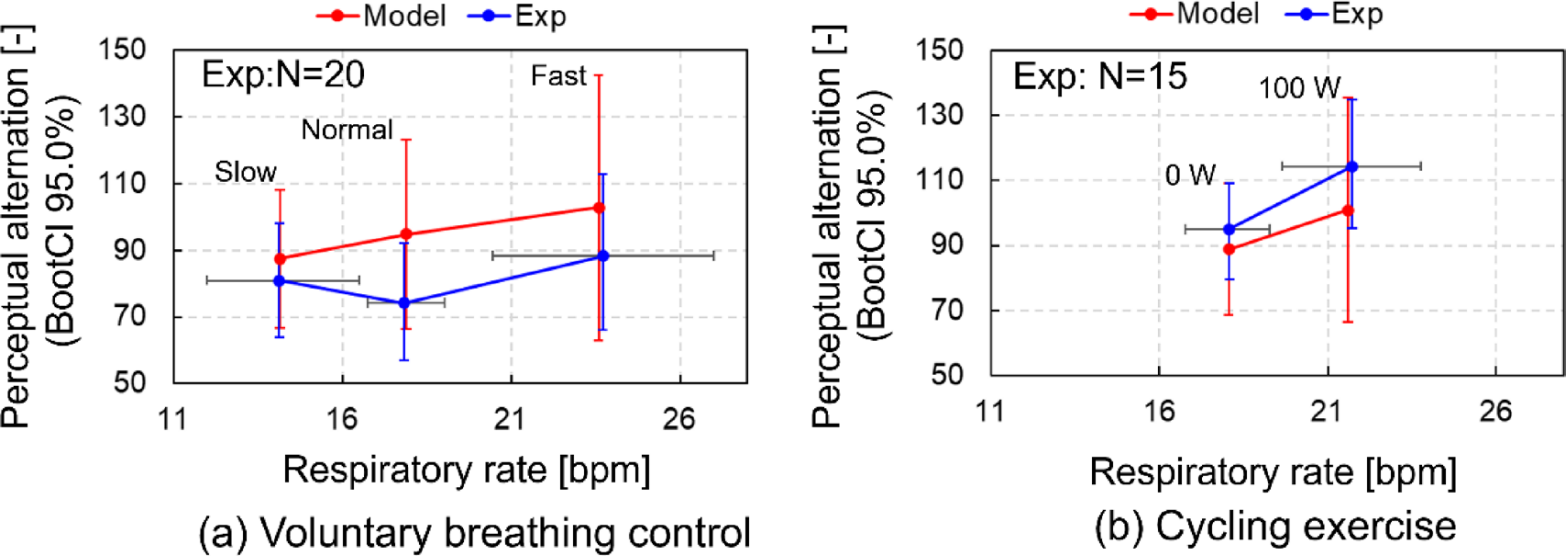
The relationship between perceptual alternation (mean values and standard deviations [mean (SD)] using the bootstrap method with 95% confidence intervals (displayed as BootCl 95.0%) and respiratory rate during voluntary respiratory control and cycling exercise. The model predicted perceptual alternation in binocular rivalry with mean (SD) using the bootstrap method with 95% confidence intervals and was compared with experimental test data conducted in previous study^16^. (a) voluntary respiratory control with slow, normal, and fast respiration, (b) cycling exercise with 0 and 100 W loads.

To investigate cognitive function in relation to brain and cardiovascular activity and variability, we focused on perceptual alternation during binocular rivalry, which is a widely used model for exploring neural and cognitive mechanisms. Figure 2 shows comparison of perceptual alternation (PA) for RR between model prediction and experimental data during voluntary respiratory control and cycling exercise. In this study, cycling exercise was assumed as an exercise that involves involuntary respiration because the participants could not voluntarily control their RR during exercise with high loads. The model predicted PA during binocular rivalry, which was compared with the experimental data obtained from a human experiment^22^. Following previous research^26^, the parameters of the pontine nuclei (pons) in the model were set to 0.97, 1.12, and 1.23 to match the mean RRs of 14.14, 17,83 and 23.72 beat/min (bpm), obtained from the experimental data of slow, normal, and fast voluntary respiratory control, respectively. The model reproduced the mean RRs in the slow- and normal-respiratory controls, with a slight difference of 0.1 bpm observed in the fast-respiratory control. For cycling exercise, the pons parameter in the model was set to 1.12 for normal respiration. The peripheral CO_2_ concentration inputs were set to −0.7 and − 7.5, and the RTN bias inputs were set to −0.35 and − 3.75, respectively. These adjustments matched the mean RRs of 18.08 and 21.73 obtained from experimental tests at 0 W and 100 W loads shown in Fig. 2(b), respectively. The model accurately replicated the mean RRs under the 0 W load, but showed a deviation of 0.1 bpm under the 100 W load. In both model prediction and test data, a higher RR increased PA under both “Slow” and “Fast” conditions during voluntary breathing control as well as under both “0 W” and “100 W” during cycling exercise (Fig. 2).

### Respiratory rate, heart rate, and arousal level during EBV

**Fig. 3:**
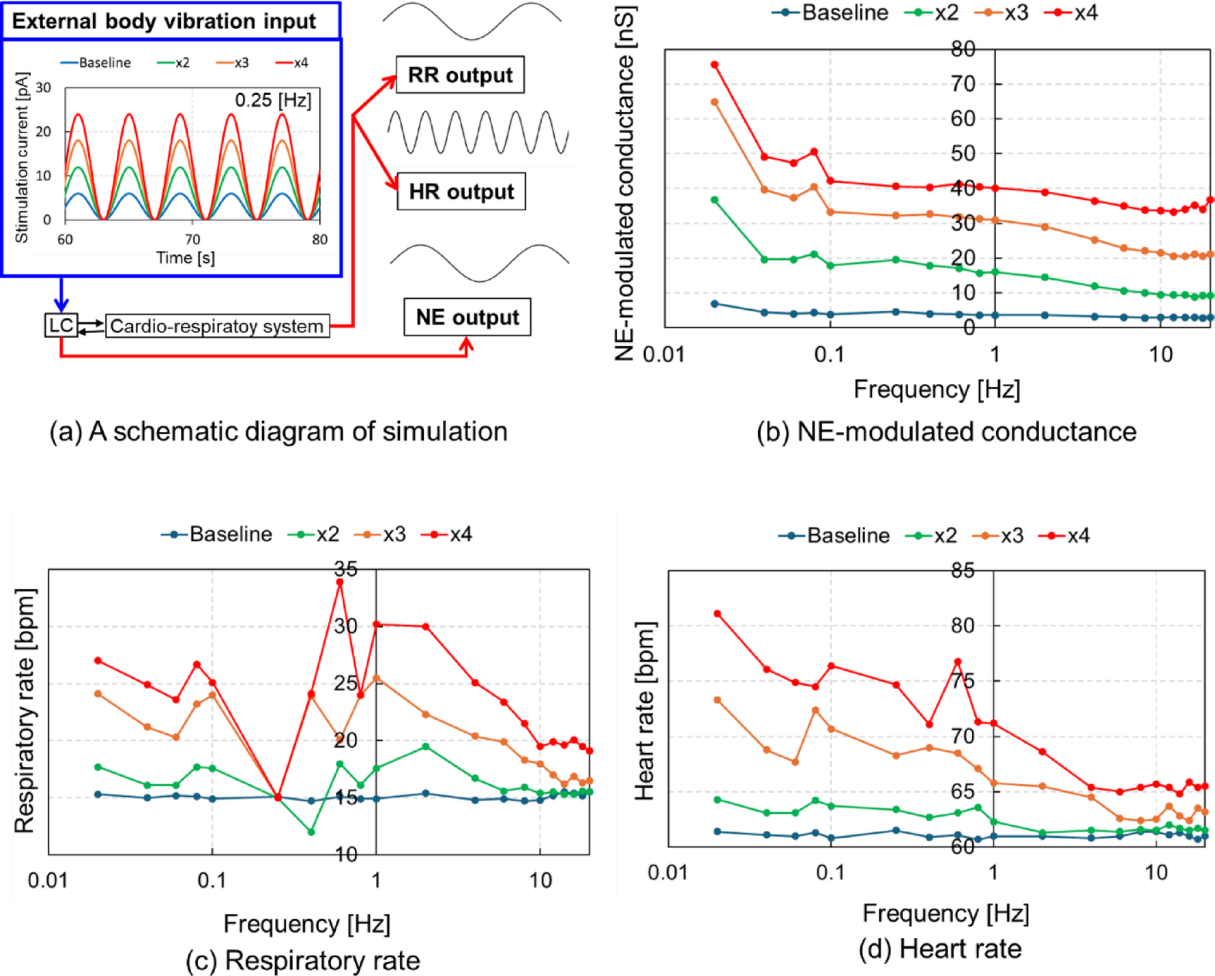
Logarithmic representation of mean values of NE-modulated conductance, RR, and HR from 700 s and 720 s for different frequencies with various amplitudes of vibrational input. (a) Schematic diagram of simulation, (b) NE-modulated conductance, (c) respiratory rate, (d) heart rate.

Figure 3 displays the logarithmic representation of NE-modulated conductance, RR, and HR across different frequencies and amplitudes of vibrational input. NE-modulated conductance, reflecting arousal level, reached its maximum at 0.02 Hz, and was lowest at 10 Hz regardless of vibration amplitude. RR showed its highest values between 0.8 and 2 Hz whereas it maintained a rate of 15 bpm at 0.25 Hz, within a range of 12 to 20 bpm, average resting RR of adults^27^, regardless of EBV amplitude. RR was also lower at a frequency ranging 10–20 Hz than at a frequency range of 0.8–2 Hz and higher at a frequency of < 0.1 Hz than that at a frequency of 0.25 Hz. HR was lower for frequencies ranging 4–20 Hz across all amplitudes and higher for frequencies < 0.1 Hz for higher amplitudes. These findings suggest that vibration frequencies < 0.1 Hz, 0.25 Hz, around 1.0 Hz, and 10.0 Hz play a crucial role in capturing the distinctive features of RR, HR, and arousal level during EBV.

### Perceptual alternation, brain variability, and heart rate variability during EBV

**Fig. 4:**
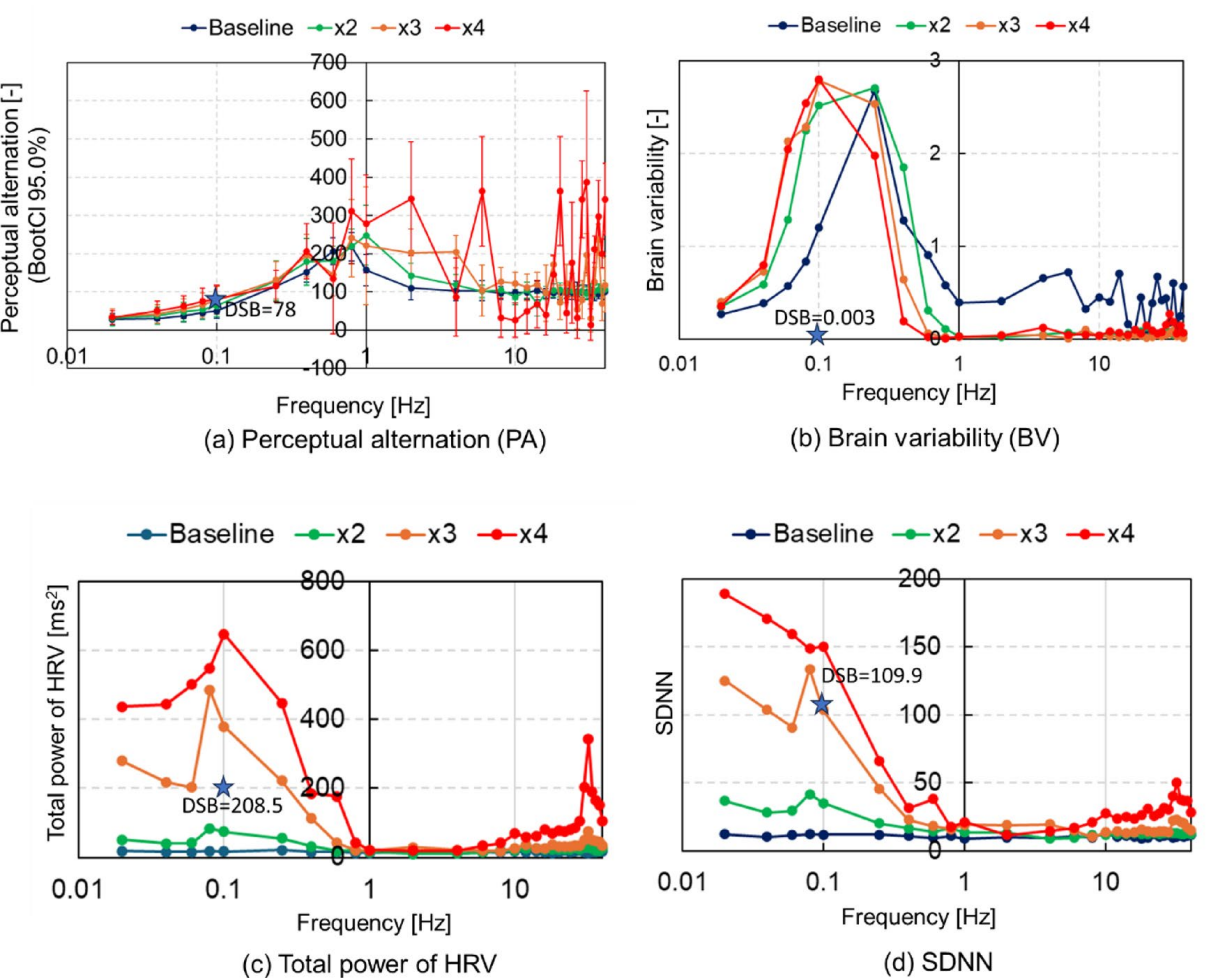
Logarithmic representation of perceptual alternation (PA), brain variability (BV), and total power of HRV for different frequencies with various amplitudes of vibrational input. (a) PA, (b) BV, (c) total power of HRV, (d) SDNN. The dark blue asterisks and numerical values indicate simulation results of DSB (deep slow breathing) control at 6 bpm.

Figure 4 illustrates the logarithmic representation of PA, BV, and HRV for different frequencies and amplitudes of vibrational input. PA was notably higher for frequencies between 0.4 and 2 Hz for all amplitudes, peaking at 6 and 20 Hz with an amplitude four times larger than that at baseline (×4). PA was lowest for frequencies < 0.1 Hz across all amplitudes and approximately at 10 Hz with a large ×4 amplitude. BV peaked at 0.1 Hz with amplitudes three and four times the baseline values (×3 and ×4), and at 0.25 Hz with amplitudes equivalent to and double the baseline (×2), whereas BV was lower at 0.02 Hz and nearly absent at frequencies > 1 Hz except the baseline. HRV exhibited its highest total power between 0.08 and 0.1 Hz, with amplitudes three and four times greater, while it decreased for frequencies between 1 and 10 Hz across all amplitudes. SDNN (standard deviation of NN intervals) was highest at 0.02 Hz with amplitudes three and four times the baseline, and decreased for frequencies ranging 1 to 10 Hz for all amplitudes. These findings highlight that vibration frequencies < 0.1 Hz, 0.25 Hz, and > 10 Hz are crucial for capturing the characteristic features of the BV, HRV, and SDNN during EBV, whereas frequencies < 0.1 Hz, around 1, and 10 Hz are critical for capturing the characteristic features of PA.

### Phase-locking between RR, HR, and LC activity during EBV

**Fig. 5:**
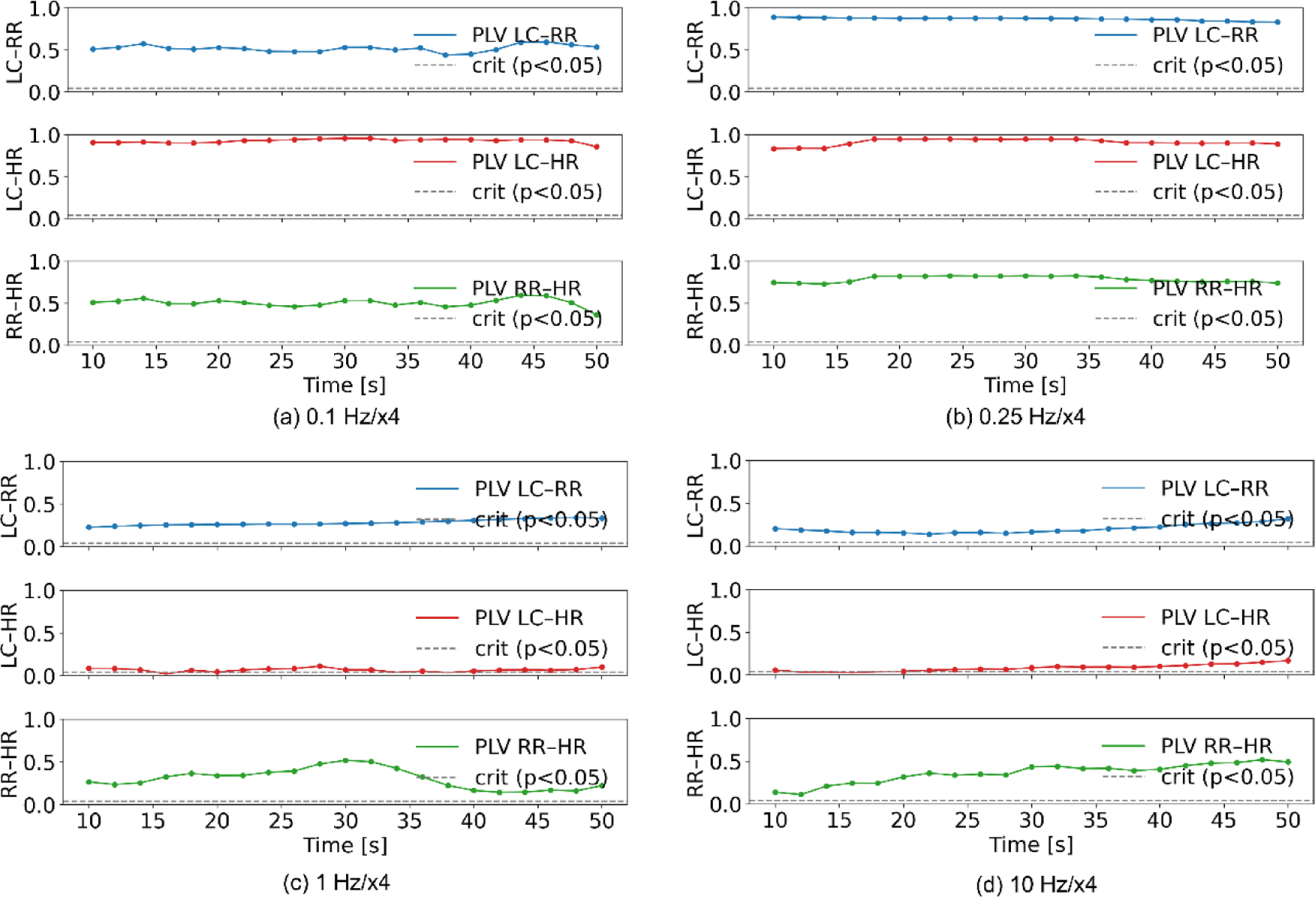
Four examples of PLV of three pairs (LC-RR, LC-HR, RR-LC) with the critical phase-locking values during the period of 60 s ranging from 660 to 720 s in EBV of 0.1, 0.25, 1.0, and 10 Hz with an amplitude of ×4. The PLV ranged from 0 to 1.0. The blue, red, and green lines with significant markers represented by solid circles indicate PLVs for LC-RR, LC-HR, RR-HR, respectively. The dotted lines represent the critical phase locking values with *p* < 0.05. (a) vibration input with a frequency of 0.1 Hz and an amplitude of ×4, (b) vibration input with a frequency of 0.25 Hz and an amplitude of ×4, (c) vibration input with a frequency of 1 Hz and an amplitude of ×4, (d) vibration input with a frequency of 10 Hz and an amplitude of ×4.

We calculated the phase-locking value (PLV) of three pairs that are LC activity (simply denoted as LC)-RR, LC-HR, and RR-HR during the period of 60 s ranging from 660 to 720 s in EBV of 0.1, 0.25, 1.0, and 10 Hz with an amplitude of ×4 using Rayleigh test as shown in Fig. 5. The critical PLV was obtained with the Rayleigh test^28^. PLV itself was computed according to the procedure described by^29^. Higher PLVs were found in LC-HR at 0.1 Hz/x4, LC-RR, LC-HR, RR-HR at 0.25 Hz/x4.

**Fig. 6:**
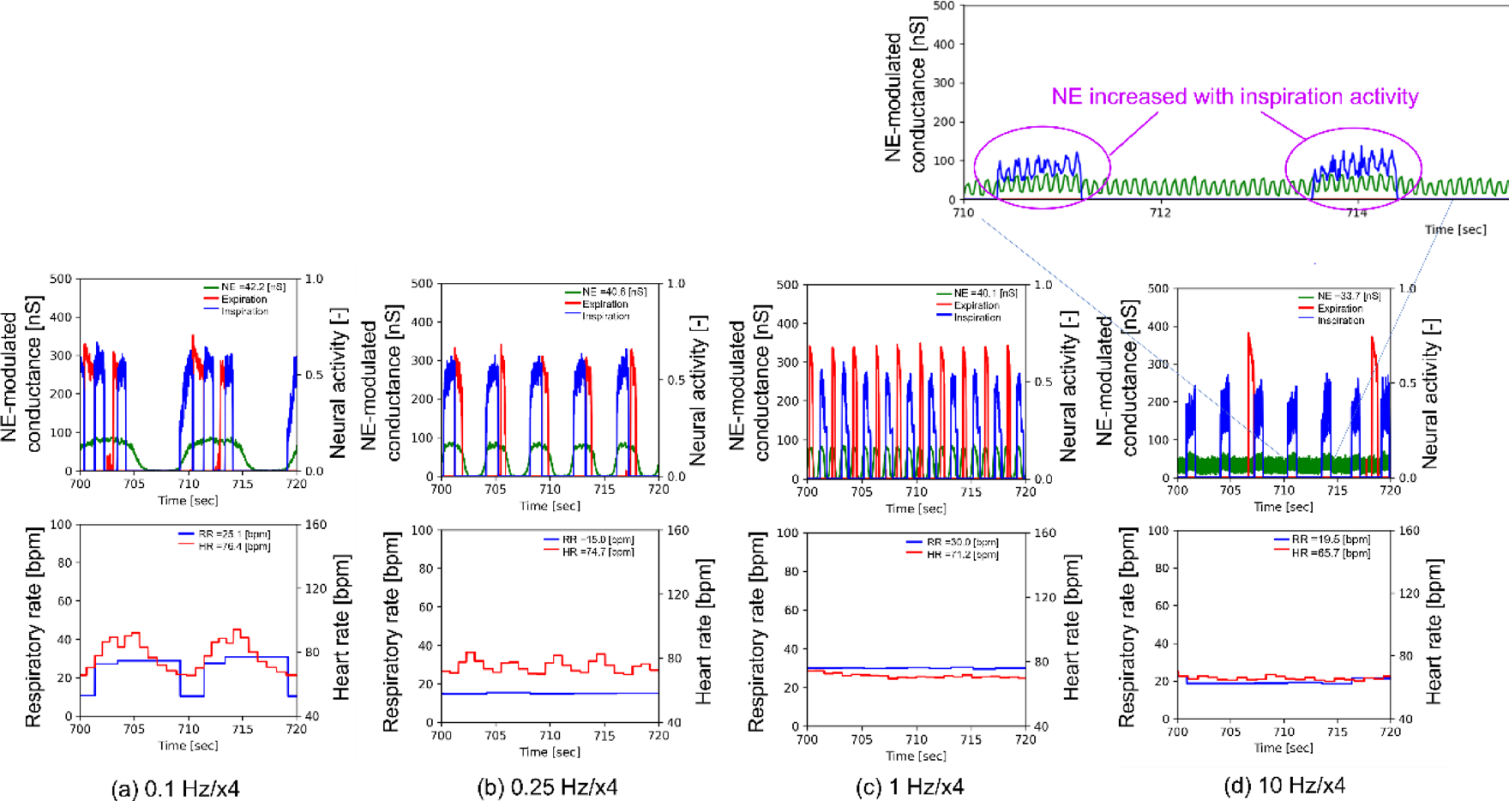
Four examples of phase locking between NE-modulated conductance (arousal level [LC activity]), RR, and HR observed during the period ranging from 700 to 720 s. In the upper part, the NE-modulated conductance, inspiration neuronal activity (diaphragm activity), and expiration neuronal activity (abdominal muscle activity) are displayed in green, blue, and red, respectively. In the lower panels, the RR and HR are shown in blue and red, respectively. (a) Vibration input with a frequency of 0.1 Hz and an amplitude of ×4, (b) vibration input with a frequency of 0.25 Hz and an amplitude of ×4, (c) vibration input with a frequency of 1 Hz and an amplitude of ×4, (d) vibration input with a frequency of 10 Hz and an amplitude of ×4. Panels (a) and (b) demonstrate 1:1 phase locking between RR, HR, and LC activity. Panel (c) demonstrates LC activity synchronized with respiratory phrenic activity (LC-respiratory entrainment) in a 2:1 phase locking manner, whereas panel (d) demonstrates PAC (phase-amplitude coupling).

Figure 6 displays four examples of phase locking between NE-modulated conductance (arousal level [LC activity]), RR, and HR observed during the period ranging from 700 to 720 s with EBV frequencies of 0.1, 0.25, 1.0, and 10 Hz, each with an amplitude of ×4. A 1:1 phase locking between the RR, HR, and LC activity was found at 0.1 Hz, whereas a 1:1 phase locking between the RR and LC activity was found at 0.25 Hz. LC activity was synchronized with respiratory phrenic activity in a 2:1 phase-locking manner at 1 Hz. Additionally, phase-amplitude coupling (PAC)^30^, which modulates the phase and amplitude of LC activity by respiratory activity as observed at 10 Hz. These results suggest that LC-respiratory entrainment contributes to fluctuations in arousal level. Furthermore, the 1:1 phase locking observed among the variability of RR, HR, and LC activity at 0.1 Hz may explain heightened arousal levels, RR, and HR observed < 0.1 Hz.

## Discussion

The cerebral cortex can be modeled using RC integrated with connectome-based connectivity based on MRI measurements^31^. Previous computational studies on artificial and spiking neural networks bearing modular structures strongly imply that modularity in biological neuronal networks can improve the performance of data classification tasks. However, whether modularity increases performance in other tasks in RC, such as motor control, signal generation, and time-series prediction tasks, remains unclear^32^. Therefore, we modeled the cerebral cortex as a recurrent neural network using RC to perform a cognitive task simulating visual perceptual alternation in binocular rivalry^24^. The reservoir model employed in this study successfully replicated the variations in PA corresponding to increasing RR observed in experimental data involving voluntary respiratory control and cycling exercises. The findings demonstrate that a higher RR may increase PA, as evidenced by increased PA under both “Slow” and “Fast” conditions during voluntary respiratory control, as well as during involuntary increases in respiration. Fast respiration reduced reaction time and movement time, leading to increased arousal ratings, whereas respiratory frequency did not differentially impact accuracy or variability across conditions in a goal-directed discrete motor task^33^. This study suggests that fast respiration increases PA levels and improves reaction time. PA predicted using this model was associated with binocular rivalry, which occurs when the eyes are presented with different stimuli, and subjective perception alternates between them. PA in binocular rivalry is related to the stability of brain dynamics^34,35^. Therefore, these simulation results suggest that the model has the potential to predict brain dynamics with changes in the RR.

We investigated the effects of EBV on cognitive performance using this model. The simulation results suggest that vibration frequencies < 0.1 Hz, 0.25 Hz, approximately 1.0 Hz, and 10.0 Hz are critical for capturing the characteristic features of change in RR, HR, and arousal level, as well as PA, BV, total power of HRV, and SDNN during EBV.

First, all indices of cardiorespiratory and brain function were low with an EBV of 10 Hz, and PA dropped between 8 and 16 Hz with the largest amplitude. Neuronal activity in the cerebral cortex at 10 Hz, with the largest amplitude of ×4, demonstrates that the cerebral cortex also remained silent at 10 Hz. These results suggest that LC neurons become inactive before the onset of each sleep spindle during 10–15 Hz high-amplitude oscillations in ECG signals in NREM sleep^36,37^, as LC activity was synchronized with EBV in this study.

Second, PA increased at frequencies 0.6 to 2 Hz, approximately 1 Hz for all amplitudes. This was because the RR was higher during EBV at approximately 1 Hz, nearly 30 bpm, with the largest amplitudes of ×4. PA was high within the frequency range of 0.6–2 Hz, supporting that musical rhythms are usually perceived to have a pulse, or basic beat, in the approximate frequency range of 0.5–4 Hz^38^ owing to entrainment of cortical rhythms to acoustic rhythms^39^. Conversely, an EBV of 1–8 Hz decreases arousal level and driving performance^40^, whereas an EBV of 1–8 Hz gradually decreases all indices with increasing frequencies, except for PA with the largest amplitude of ×4 and BV with the baseline amplitude in the simulation results. Neuronal activity in the cerebral cortex at 1 Hz, with the largest amplitude of ×4 demonstrates that the cerebral cortex exhibited significantly decreased variability at 1 Hz. Therefore, this model has the potential to capture the characteristic features of brain dynamics during EBV exposure.

Third, the variability in neural signals is linked to cognitive capacity. BV appears to increase during task performance compared with that at rest in younger, faster-performing adults, whereas older and slower-performing adults exhibit less differentiation in brain variability across experimental conditions^16^. BV peaked at 0.1 Hz with three and four times larger amplitudes, and at 0.25 Hz with smaller amplitudes at baseline and double the baseline (×2). Neuronal activity in the cerebral cortex at 0.1 and 0.25 Hz, with the largest amplitude of ×4, demonstrates increased variability. To the best of our knowledge, no study has investigated the relationship between vibration frequencies < 1 Hz and cognitive performance, although negative effects of vibration frequencies > 1 Hz on performance have been reported^11,41^. The high-HRV group performed better on executive-function tasks in reaction time and accuracy parameters^18^. Total power of HRV peaked at 0.1 Hz across all amplitudes, whereas it was lower at 0.25 Hz with smaller amplitudes at baseline and double the baseline. Therefore, EBV at 0.1 Hz has a greater potential to enhance cognitive performance than other frequencies.

Fourth, Ujjayi breathing, a victorious breathing technique in yoga, involves respiratory control at 1 bpm (0.02 Hz), shows a relaxing effect, reduced stress, increased peacefulness, and the feeling of being at ease/leisure^42^. The resting HR was 69 bpm in the control breathing group at 6 bpm, whereas it was 75 bpm in the Ujjayi breathing group at 1 bpm^43^. EBV at 0.02 Hz with the largest amplitude of ×4 increased HR, arousal level, and SDNN compared with EBV at 0.1 Hz, whereas it decreased PA, BV, and total power of HRV. SDNN reflects all long-term components and circadian rhythms responsible for variability over the recording period^44^. EBV at 0.02 Hz with the largest amplitude of ×4 has the potential to increase arousal level and HRV. During development from childhood and mid-adulthood, brain signal variability increases, showing a negative correlation with reaction time variability and a positive correlation with accuracy^17^. EBV at 0.1 Hz increased BV but decreased PA, despite an increase in RR at 0.1 Hz. Because RR influences PA and improves reaction time^33^, BV is hypothesized to relate more to accuracy or cognitive flexibility, facilitating adaptive adjustments in thoughts and behaviors in response to changing environmental demands^15^.

Phase locking of oscillators is a typical phenomenon of self-organization in physical, chemical, and biological systems. RR maintained a rate of 15 bpm at 0.25 Hz, regardless of EBV amplitude, attributed to LC respiratory entrainment with 1:1 phase locking^23^, whereas PAC, which modulates the phase and amplitude of LC activity by respiratory activity, was observed at 10 Hz (Fig. 6d). The increase in BV at 0.1 Hz and 0.25 Hz is likely attributed to LC activity (green lines) synchronized with variability of HR (red lines) and RR (blue lines) in a 1:1 phase locking manner (Fig. 6a, b). LC activity was entrained by fluctuations of HR and RR at 0.1 Hz, whereas it was entrained solely by fluctuation of HR at 0.25 Hz, potentially explaining the higher BV observed at 0.1 Hz than at 0.25 Hz. The cardiorespiratory synchronization is found more frequently during arousal than during neutral states^45^. Synchronization among the LC, cardiac, and respiratory systems is hypothesized to elevate brain and heart variability, thereby enhancing cognitive performance. The simulation results suggest that such synchronization can be achieved with EBV at 0.1 Hz by increasing the amplitude.

Meditation, which includes conscious regulation of respiration, enhances executive functioning and attention sustainability^46^. Moreover, meditation and music listening significantly enhance subjective memory function and objective cognitive performance in adults with subjective cognitive decline^47^. Mindfulness meditation also enhances short-term cognitive performance without prior practice^48^. Diaphragmatic breathing with an average RR of 4 bpm improves cognitive performance and reduces stress-related physiological effects in healthy adults^49^. Yoga meditation at a RR of 5.4 bpm increases HR and HRV, especially the low-frequency component^50^. Therefore, meditation with a slow RR of < 6 bpm (0.1 Hz) enhances cognitive performance and HRV. In our simulation, we modeled respiratory control at a slow RR of 6 bpm by setting the pons parameter to 0.43. Figure 4 illustrates simulation results (indicated by dark blue asterisks and numerical values) for DSB control at 6 bpm. The simulation results demonstrated that respiratory control increased HRV but not BV, whereas EBV at 0.1 Hz increased BV and HRV. As meditation involves various procedures beyond DSB control, simulating respiratory control may not adequately replicate meditation with deep slow RR. Further studies are needed to refine the model’s ability to simulate meditation accurately. However, EBV at 0.1 Hz with larger amplitudes holds promise for enhancing cognitive performance similarly to meditation. Cognitive training, physical activity, and bilingual experience are currently utilized in various interventions^15^. Physical activity interventions include aerobics, material art, yoga, and meditation^51^. Considering EBV at 0.1 Hz with larger amplitudes as a potential addition to these interventions warrants further investigation. Confirmation of the simulation results’ validity should involve comparisons with experimental studies on EBV across frequencies ranging from 0.02 to 1 Hz and various amplitudes.

This study has a few limitations. First, we used the assumed current values of −3, −6, −9, and − 12 [pA], which correspond to baseline, ×2, ×3, and ×4 in the figures, to represent the amplitudes of vibration inputs to the LC. Further neurophysiological studies are necessary to determine the magnitude of the electrical stimulus to the LC when the human body sustains external vibrations. Second, the model could not simulate the respiratory control of RRs < 4 bpm by adjusting the pons parameter. When the pons parameter was set to 0.3, RR and HR were 4.2 and 155.6 bpm, respectively. Although we decreased the pons to < 0.3, the RR did not decrease to < 4 bpm. Further studies on the modeling of diaphragmatic breathing are needed to reproduce meditation with deep slow RR. Third, our human experiment recruited only male participants primarily due to practical constraints related to electrode and chest-belt attachment. Therefore, the possibility of sex differences in the results cannot be excluded, and future studies should explicitly include female participants to examine potential sex differences.

Finally, human cognitive processes can be divided into three domains—perceptual, memory, and executive-control systems—and perceptual alternation represents only one aspect of perceptual function. In this study, we used a reservoir model to reproduce the switching dynamics of binocular rivalry and to quantify brain variability. This variability appears to act as the driving force behind perceptual alternation and as the flexible switching mechanism that governs both the memory and executive-control systems. We plan to further model and evaluate how brain variability affects cognitive processes.

In conclusion, this study utilized a RC model to successfully replicate variations in PA linked to changes in RR observed during voluntary control and exercise conditions. The findings highlight how increased RR enhances PA and influences cognitive functions such as reaction time and arousal level. Moreover, the model’s ability to predict brain dynamics under varying environmental conditions, particularly with respect to EBV, underscores its potential in neurophysiological research and clinical applications. Future studies should focus on refining the model’s parameters and validating its predictions through comprehensive experimental studies across different frequency and amplitude ranges of EBV.

## Methods

###  Modeling of brain–cardio–respiratory dynamics

Figure 1 illustrates a computational model for predicting RR, HR, arousal level, and cognitive performance following external stimulation. The model includes interactions among the LC-NE, cardiorespiratory systems, and the cerebral cortex. We modeled the amygdala, RTN, RVLM, caudal ventrolateral medulla (CVLM), and AMB using 20, 40, 30, 30, and 30 Hodgkin–Huxley (HH)-type spiking neurons. Baroreceptor-related neurons in NTS and IE phase-spanning neurons included 20 and 30 sets of excitatory and inhibitory HH-type neurons, respectively. Chemoreceptors in the NTS include two groups of 30 HH-type neurons: one receives CO_2_ signals from the lungs and excites or inhibits the amygdala, whereas the other comprises second-order peripheral chemoreceptors that have direct projections to the RTN, preI/I (pre-BotC) of the respiratory center, and RVLM. Since the number of HH-type spiking neurons may influence the simulation results^52^, we performed a sensitive analysis and determined the number of the HH-type neurons mentioned above^23^. The amygdala was assumed to inhibit both NTS (Chemo)^53^ and NTS (PSR)^54^, whereas NTS (Chemo) was assumed to send excitatory projections to the amygdala^55^.

Each neuron in the amygdala, RTN, NTS, RVLM, CVLM, AMB, and IE can be fundamentally represented using the following Eq. (1):

1 $$\:C\frac{dV}{dt}=-{\stackrel{-}{g}}_{K}{m}_{K}^{4}\left(V-{E}_{K}\right)-{\stackrel{-}{g}}_{Na}{m}_{Na}^{3}{h}_{Na}\left(V-{E}_{Na}\right)-{g}_{L}\left(V-{E}_{L}\right)-I-\sqrt{2D}{\upxi\:}\left(t\right).$$

The LC was modeled using a 15 × 15 excitatory neural network with an HH-type equation, as the follows Eqs. (2)(3):

$$\:C\frac{dV}{dt}=-\phi\:{g}_{L}\left(V-{E}_{L}\right)-{\stackrel{-}{g}}_{K}{m}_{k}^{4}\left(V-{E}_{K}\right)-{\stackrel{-}{g}}_{Na}{m}_{Na}^{3}{h}_{Na}\left(V-{E}_{Na}\right)-\text{I}-\sqrt{2D}{\upxi\:}\left(t\right)$$

2 $$\:\:\:\:\:\:\:\:\:\:\:\:\:\:\:-\phi\:{I}_{syn}^{E}-{I}_{NE}^{I}-{I}_{gap}$$

3 $$\:\:\:\:\:\:\phi\:=1-\frac{1}{1+{\left({s}_{1/2}/s\right)}^{{h}_{s}}}\:$$

The modulatory dynamics for $\:{g}_{NE}$ are described using the second-order delay system, as the following Eq. (4):

4 $$\:\:\:\:{\tau\:}_{0}\dot{y}=-y+\sum\:_{j}\delta\:\left(t-{t}_{j}\right),\:\:\:\:\:\:\:{\tau\:}_{1}{\dot{g}}_{NE}=-{g}_{NE}+y\:,\:\:\:\:\:\:\:\:$$

where $\:{t}_{j}$ is the *j* th firing spike, $\:\delta\:$ is the delta function, and $\:{g}_{NE}$ corresponds to the NE-modulated conductance released into the surroundings. $\:{I}_{NE}^{I}={g}_{NE}^{0}{g}_{NE}\left(V-{V}_{syn}^{I}\right).$

The electrophysiological model of cardiac muscle activity is described by the following diffusion Eq. (5):

5 $$\:\:\:\:\:\dot{v}=D{\nabla\:}^{2}v+c\left(v\left(v-a\right)\left(1-v\right)-w\right),\:\:\:\dot{w}=b\left(v-dw\right),\:c=\left\{\begin{array}{c}{c}_{1S}\left(\dot{v}\ge\:0\right),\\\:{C}_{2S}\left(\dot{v}<0\right),\end{array}\right.$$

where $\:v$ is the membrane potential of cardiac muscle cells, and $\:w$ is the recovery variable. $\:{c}_{1S}={G}_{c}{\stackrel{-}{c}}_{1S},\:{c}_{2S}={G}_{c}{\stackrel{-}{c}}_{2S}$. We hypothesized that $\:{G}_{c}$ and *b* would be determined by the neuromodulation of NE and Ach, as shown in the following Eq. (6), based on Hill’s Eq. (7).

6 $$\:\:\:\:\:{G}_{c}=\left({c}_{Max}-{c}_{Min}\right)*H\left({p}_{NE}\right)+{c}_{Min},\:\:b=\left({b}_{Max}-{b}_{Min}\right)*H\left({q}_{Ach}\right)+{b}_{Min}$$.

7 $$\:\:\:\:\:H\left({p}_{NE}\right)=\frac{1}{\left(1+{\left(\frac{{p}_{{NE}_{mid}}}{{p}_{NE}}\right)}^{{\alpha\:}_{NE}}\right)},\:\:H\left({q}_{Ach}\right)=1-\frac{1}{\left(1+{\left(\frac{{q}_{{Ach}_{mid}}}{{q}_{Ach}}\right)}^{{\alpha\:}_{Ach}}\right)}.$$

Parameters of these equations used in each model are listed in Table 1 including a column to explain the sources of all parameter values: [1] calibrated [2] from calibrated values reported in other models.

The detailed description and all parameter values of the LC-NE and cardiorespiratory systems was provided in our previous study^23^.

**Table 1:** Lists of all parameters used in this study with the sources of their values and the references. [1] calibrated, [2] from calibrated values reported in other models.

Models	Parameters	Sources	References
Respiratory center and system	Membrane capacitance: C Conductance: $\:{g}_{L},{g}_{I,}{g}_{E},{g}_{Kdr},{g}_{NaP},{g}_{AD}$ Reversal potential: $\:{E}_{L},{E}_{I,}{E}_{E},{E}_{K},{E}_{Na}$ Time constant: $\:{\tau\:}_{m2},\:\:{\tau\:}_{m3}$, $\:{\tau\:}_{m4}$ Conversion coefficient: $\:{k}_{1},\:{k}_{2}{,\:k}_{3},{k}_{4},{k}_{d},{k}_{a}$ Pressure: $\:{P}_{m},\:{P}_{L0}{,\:P}_{w},{p}_{c}^{0},\:{P}_{o}^{0}{,P}_{S0},{\varDelta\:P}_{c}$ Partial pressure including hemoglobin: $\:\sigma\:,\:{\sigma\:}_{C},L,\:{K}_{T},{K}_{R},\:{V}_{C},\:{C}_{u},\:{T}_{h},\:{r}_{2},{l}_{2},{z}_{0},\:H$ Airway resistance: R Lung elastance: E Heart period: $\:{T}_{L}$ Volume of the lung capillaries: $\:{V}_{C}$ Acceleration rate of the chemical reaction: $\:\delta\:$ Diffusion capacity of CO_2_: $\:{D}_{C}$ Diffusion capacity of O_2_: $\:{D}_{O}$	[2] [2] [2] [1] [2] [2] [2] [2] [1] [1] [1] [1] [1] [1]	^25^ ^25^ ^25^ ^25^ ^25^ ^25^ ^25^
Cardiac system	Cardiac muscle: $\:{\stackrel{-}{C}}_{1S},\:{\stackrel{-}{C}}_{2S}$,D Neuromodulation of NE and Ach based on Hill’s equation: $\:{c}_{Max},\:{c}_{Min,}{b}_{Max},{b}_{Min},{P}_{{NE}_{mid}},{q}_{{Ach}_{mid}},{\alpha\:}_{NE},{\alpha\:}_{Ach}$	[2] [1]	^56^
RVLM, CVLM, AMB, IE	Membrane capacitance: C Conductance: $\:{g}_{L},{\stackrel{-}{g}}_{KS},{\stackrel{-}{g}}_{NaP}$ Reversal potential: $\:{E}_{L},{E}_{K},{E}_{Na}$ Time constant: $\:{\stackrel{-}{\tau\:}}_{KS}$ Noise strength: D	[2] [2] [2] [2] [1]	^57^ ^57^ ^57^ ^57^
Amygdala, NTS, RTN, LC-NE system	Membrane capacitance: C Conductance: $\:{g}_{L},{\stackrel{-}{g}}_{K},{\stackrel{-}{g}}_{Na}$ Reversal potential: $\:{E}_{L},{E}_{K},{E}_{Na}$ Time constant: $\:{\stackrel{-}{\tau\:}}_{KS}$ CO_2_ modulation: $\:{s}_{1/2},{h}_{s},{g}_{gap},{g}_{NE}^{0},{V}_{sym}^{l}$ Time constant: $\:{\tau\:}_{o},\:{\tau\:}_{1}\:$	[2] [2] [2] [2] [2] [2]	^58^ ^58^ ^58^ ^58^ ^59^ ^60^
Cerebral cortex	Spectral radius: $\:\rho\:$ Activation gain: $\:\alpha\:,\beta\:$ Time constant: $\:\tau\:$	[1] [1] [2]	^61^

We modified the RTN bias from − 5 to −1 and vital capacity from 5.6 to 4.8 L to reproduce normal respiration with maximum RR of around 30 bpm and minute ventilation of approximately 8.0 L/min in this study^62^. Voluntary RR is adjusted by a parameter (called pons) of the tonic drive in the pons following previous reserach^26^, whereas active expiration with exercise load is adjusted by peripheral CO_2_ concentration input to the NTS chemo and RTN bias input. Vibrational input was provided directly to the LC.

The cerebral cortex was modeled as a recurrent neural network using an echo state network (ESN), a typical RC model with a sparsely connected hidden layer, to output the side of the image that was perceived as input from the left and right eyes, to perform a cognitive task simulating visual perceptual alternation in binocular rivalry^24^. Each neuronal activity in the ESN corresponded to the behavior of neuron groups averaged and abstracted from the cerebral cortex. In the ESN, the connectivity and weights of the hidden-layer (reservoir) neurons were fixed (not trained) and randomly assigned. The output layer’s (readout) neurons have weights that are trainable and can be learned such that the network can produce or reproduce specific temporal patterns. An ESN can make most of the echo-like dynamics of a reservoir, allowing it to effectively capture and replicate temporal patterns in sequential inputs and read out input features. A study that investigated how the performance of an ESN changes as the reservoir size is increased^63^. From their results, the memory capacity (MC) of an ESN with 400 neurons constructed based on the human whole-brain connectome is MC(400) = 25. They investigated MC with significantly larger neuron numbers, up to 1,750 neurons. It appears that MC asymptotically approaches a certain maximum value: MC(INF) = 30. Therefore, an ESN with 400 neurons has enough MC, reaching 80% of its maximum capacity. We set the number of neurons, N, in the reservoir to 400 by balancing the trade-off between achieving the accuracy needed to fulfill the simulation’s objectives and keeping computation time as short as possible.

We set inputs with dimensions of M = 2 to [1,1] ^T^ corresponding to [input to the left eye, input to the right eye]^T^ and set outputs with dimensions of K = 2 to [left image perception, right image perception] ^T^ determined depending on which side of the image is largely perceived according to Eq. (9). The ESN model simulates the integration of visual information from the left and right eyes into the cerebral cortex using the follows Eqs. (8)-(10):

8 $$\:\tau\:\frac{dx\left(t\right)}{dt}=-x\left(t\right)+tanh\left\{k({W}^{in}u\left(t\right)+{W}^{r}x\left(t\right))\right\}$$

9 $$\:y\left(t\right)={W}^{out}x\left(t\right)$$

10 $$\:s={arg}_{index}max\:y\left(t\right)$$

where $\:x\in\:{R}^{N}$ is an internal state vector of ESN, $\:u\in\:{R}^{M}$ is an input vector, and $\:{W}^{in}\in\:{R}^{N\times\:M}$, $\:{W}^{r}\in\:{R}^{N\times\:N}$, and $\:{W}^{out}\in\:{R}^{K\times\:N}$ are input, internal, and output weight matrices, respectively. Equation (10) indicates that *s* receives the index of maximum element *y(t)*. The parameter k represents the activation gain, which is determined using the following Eq. (11)^24^:

11 $$\:k=\alpha\:\times\:NE+\beta\:$$

where $\:\alpha\:=-0.07,\:\beta\:=3.65$ based on parameter tuning. NE denotes the amount of NE that diffuses from the LC. Previous studies on the measurements and analyses of biological neurons demonstrated that an increase in the mean firing rate of the whole brain leads to an increase in synaptic background noise received by single neocortical neurons, resulting in linear firing rates for inputs^64,65^. Therefore, we assumed that the firing rates of cortical neurons for NE inputs would be linear, as shown in Eq. (11), because an increase in NE enhances the mean firing rate of the whole cortex and subsequently increases the synaptic background noise. We performed a sensitivity analysis to examine the relationship between the activation gain *k*, the spectral radius $\:\rho\:$, and the RC’s responses, which were obtained using Lyapunov exponent^66^. When we set the spectral radius as 0.95 and the RC output the NE under a normal RR, the $\:\alpha\:$ and $\:\beta\:$ parameters were engineered so that respiration drives the system back and forth between both the chaotic region—where perceptual switches are expected to occur readily—and the input-dependent region, which corresponds to a perception-fixed mode^24^. As a result, the *α* and *β* parameter values were selected to be −0.07 and 3.65, respectively, to adjust *k* to have a range from 0.5 to 2.6 for the NE values with a range from 15 to 45. PSP for the cerebral cortex can be computed by the mean value of all reservoir neuron outputs (internal state vector x(t) of ESN shown in Eq. (8)) multiplied by a parameter on feedback from the cortex to LC called lcVPSP (= 0.1). This is because LC-NE receives information from many brain regions. The LC-NE circuit overall integrates information from, and broadcasts to, many brain regions, consistent with its primary role in regulating brain states^67^.

The weight matrices $\:{W}^{in}$ and $\:{W}^{r}$ include non-zero components of random numbers obeying uniform distribution ranging from − 1 to 1. The density of Erdos-Renyi is the connection probability between each neuron and determines the number of nonzero components (connection sparsity) in the internal weight matrix $\:{W}^{r}$, whereas the spectral radius is the maximum absolute value of the eigenvalues of the matrix $\:{W}^{r}\:$affecting the chaoticity of the reservoir. The density was set to 0.05. $\:s$ denotes the output of the readout and is given as 0 or 1 where the right and left eyes are predominant, respectively. The simulation time step was set at 0.1 ms to be used with simulations of the cardiorespiratory system, and the time constant of the reservoir $\:\tau\:$ was set at 375 ms according to a previous study^61^.

The interaction between the LC-NE and cardiorespiratory systems was implemented using C + + language, whereas the interaction between the LC-NE system and the cerebral cortex was implemented using Python 3.8. Each parameter value listed in Table 1 was used as an initial condition, and the differential Eq. (1)(2)(4)(5)(8) were numerically integrated using the Euler method. Bidirectional data communication of the NE and PSP between the LC and the cerebral cortex was accomplished using the open-source universal messaging library of ZeroMQ (zmq).

###  Human experiment

Experimental tests were conducted to investigate the relationship between respiratory speed and visual PA during binocular rivalry^22^.

Twenty healthy male participants (mean age: 24.5 ± 2.8 years) and fifteen healthy male participants (mean age: 24.3 ± 2.5 years) took part in voluntary respiratory control and cycling exercise, respectively. The study protocol was approved by the Ethics Committee of the University of Tokyo (approval number: 18–247). Written informed consent was obtained from all participants prior to their participation in the study, in accordance with the Declaration of Helsinki.

For the binocular rivalry task, visual stimuli comprising dichoptic orthogonal gratings as shown in Fig. 1 were presented for 6 min via a head-mounted display (VIVE Pro Eye, HTC, Japan).

Participants were instructed to indicate their dominant perception—whether the image presented to the left or right eye—by pressing one of two buttons. The timing of perceptual alternations was recorded. A respiration belt positioned around the torso measured thoracic expansion and contraction at 500 Hz, allowing for extraction of RR. In parallel, ECGs were recorded at 500 Hz, with R-peaks subsequently extracted for heart rate analysis. The experiment commenced following an initial training session.

Two conditions were employed to investigate the relationship between breathing patterns and rivalry dynamics: a rest condition, in which participants maintained their natural RR and voluntarily controlled slow, normal, and fast RRs, and a cycling condition, during which participants engaged in exercise with a 100 W load.

The distribution of the respiratory frequency was estimated using the bootstrap method with 95 confidence intervals, and the mean (SD) of the distribution was calculated. The respiratory phase was obtained using the Hilbert transformation. PA was also estimated as the mean (SD) of the switching number, obtained by counting the number of times a participant pressed a button for > 6 min at each respiratory speed using the bootstrap method with 95 confidence intervals. We determined the relationship between the respiratory phase and PA using these test data.

### Computational experiment design

First, the model was validated against changes in PA during voluntary respiratory control at slow, normal, and fast RRs, and during cycling exercise with two loads of 0 and 100 W. For voluntary respiratory control, the pons parameter was adjusted to reproduce the mean RRs of slow, normal, and fast respiratory measured in experimental tests using twenty human participants. For cycling exercise, the pons parameter was set to a constant value for normal respiratory, and the peripheral CO_2_ concentration and RTN bias inputs were adjusted to the mean RRs obtained from experimental tests of cycling exercise at 0 W and 100 W loads using fifteen human participants.

The model predicted the PA using the mean (SD) during voluntary respiratory control and cycling exercises. The dynamics of the RC model fluctuated depending on the weight matrices of the input and internal states. Thus, we calculated the dynamics by setting the random seed values of the weight matrices in the range of 0–9. The respiratory phase was calculated using pCO_2_ obtained from the cardiorespiratory system. After the phase was shifted such that the mean pCO_2_ value was zero in the total simulation period, it was adjusted to the respiratory phase from the experimental test using Hilbert transformation. Subsequently, PA was estimated as the mean (SD) of the switching number in each respiratory phase output from the RC model for 720 s, with random seed values in each simulation condition, using the bootstrap method with 95% confidence intervals. Each respiratory phase ranged from $\:\theta\:-\frac{\pi\:}{4}$ to $\:\theta\:+\frac{\pi\:}{4}$, where $\:\theta\:$ of 100 was obtained from -$\:\pi\:$ to $\:\pi\:$ in each simulation condition.

Second, we used the model for autonomic respiratory simulation under the following EBV conditions: the parameter of the pons was set to 1.0 to simulate normal RR, and the resulting RR, HR, and NE-modulated conductance without any stimulation (corresponding to the resting state) were 15.1, 60.8, and 0.79, respectively. The RR and HR values were nearly identical to those in healthy adult males in the resting state^27,68^. The simulation condition included 80 inputs to simulate EBV via sinusoidal waves with four amplitudes (−3, −6, −9, and − 12 [pA]) and 20 frequencies (0.02, 0.04, 0.06, 0.08, 0.1, 0.25, 0.4, 0.6, 0.8, 1.0, 2.0, 4.0, 6.0, 8.0, 10.0, 12.0, 14.0, 16.0, 18.0, and 20.0 [Hz]) over a time period of 720 s. A duration of 720 s is much longer than the 6-minute perceptual-alternation experiment in psychology and provides a sufficient number of samples for discussing transition probabilities and related measures. Vibrational inputs to mammalian bodies are known to stimulate the vestibular sensory organs and induce LC activity that is physically synchronized with the original vibrational inputs^69^. However, the mechanism through which the amplitudes of the vibrational inputs are transmitted to the LC is unknown. Therefore, we used the current value inputs to HH-type neurons to represent the amplitudes of the stimulation input to the LC. All amplitudes represent excitatory stimulation, which increases with the absolute value. In this study, we defined the amplitude of −3 [pA] as the baseline and subsequently depicted − 6, −9, and − 12 [pA] as ×2, ×3, and ×4 the baseline, respectively.

We calculated the RR, HR, and NE-modulated conductance from the LC to the cortex, which represents the arousal level in addition to activity of inspiration and expiration neurons. The RR, HR, and NE-modulated conductance were calculated as average values during the period ranging from 700 to 720 s in the autonomic respiratory simulations. We investigated the relationship between the EBV inputs and model outputs of arousal level, RR, HR, PA, BV, HRV total power, and SDNN using the obtained simulation results.

The PA was calculated using the method mentioned earlier. BV was calculated using the mean squared successive difference (MSSD, $\:{\delta\:}^{2}$) defined as the following Eq. (12):

12 $$\:{\delta\:}^{2}=\frac{{\sum\:}_{i=1}^{n-1}{\left({x}_{i+1}-{x}_{i}\right)}^{2}}{n-1}$$

MSSD involves squaring the difference between time point $\:i$ and time point $\:i+1$ $\:\left({x}_{i+1}-{x}_{i}\right)$, and averaging these values across the entire brain signal time course^15^. MSSD is a method used for computing BV from resting-state BOLD signal variability^70^. We plotted the time histories of activities of 400 neurons in the cerebral cortex (RC model) and calculated MSSD for each neuronal activity. The average value of all MSSD in the cerebral cortex was defined as the BV.

We also calculated the total power of the HRV and SDNN as measures of heart rate variability. The total power of HRV is a global index, representing the sum of energy in the ULF (ultra low frequency), VLF (very low frequency), LF (low frequency), and HF (high frequency) bands over 24 h, and in the VLF, LF, and HF bands for short-term recordings^71^. SDNN is another global index of HRV, calculated as the standard deviation of all NN intervals, reflecting long-term components and circadian rhythms influencing variability during the recording period^44^. We used pyHRV^72^ to calculate the SDNN and total power of HRV.

All simulations were performed on a computer equipped with an Intel Xeon Gold 6242 (16 C/2.8 Ghz) and 384 GB Memory.

## Data Availability

The source codes used to generate the results in this paper are available at https://osf.io/gjsb7/?view_only=e51bb3f3e0084cb1a9a8988cefac5b0a.
